# Flexibility of internal attention: divergent spatiotemporal profiles for feature and object cueing

**DOI:** 10.1093/cercor/bhag110

**Published:** 2026-09-17

**Authors:** Xiaoshu Lin, Kristina Pultsina, Simo Monto, Chaoxiong Ye, Tiina Parviainen

**Affiliations:** Department of Psychology, University of Jyväskylä, Ruusupuisto (RUU Building), Alvar Aallon katu 9, FI-40014 Jyväskylä, Finland; Centre for Interdisciplinary Brain Research, University of Jyväskylä, Kärki Building, Mattilanniemi 6, FI-40014 Jyväskylä, Finland; Department of Psychology, University of Jyväskylä, Ruusupuisto (RUU Building), Alvar Aallon katu 9, FI-40014 Jyväskylä, Finland; Centre for Interdisciplinary Brain Research, University of Jyväskylä, Kärki Building, Mattilanniemi 6, FI-40014 Jyväskylä, Finland; Department of Psychology, University of Jyväskylä, Ruusupuisto (RUU Building), Alvar Aallon katu 9, FI-40014 Jyväskylä, Finland; Centre for Interdisciplinary Brain Research, University of Jyväskylä, Kärki Building, Mattilanniemi 6, FI-40014 Jyväskylä, Finland; Department of Psychology, University of Jyväskylä, Ruusupuisto (RUU Building), Alvar Aallon katu 9, FI-40014 Jyväskylä, Finland; School of Education, Anyang Normal University, No. 436 Xian'ge Avenue, Anyang, Henan Province 455000, China; Department of Psychology, University of Jyväskylä, Ruusupuisto (RUU Building), Alvar Aallon katu 9, FI-40014 Jyväskylä, Finland; Centre for Interdisciplinary Brain Research, University of Jyväskylä, Kärki Building, Mattilanniemi 6, FI-40014 Jyväskylä, Finland

**Keywords:** alpha oscillations, internal attention, magnetoencephalography (MEG), spatiotemporal dynamics, working memory

## Abstract

Internal attention efficiently prioritizes information in working memory (WM), yet it remains unclear whether it reflects a unified, domain-general mechanism, or a flexible set of operations tailored to the format of selected information. Using magnetoencephalography (MEG), we characterized the spatiotemporal alpha-band dynamics underlying feature-based (color, orientation) and object-based (left, right) retro-cues that direct internal attention within WM. Behaviorally, both cue types improved performance relative to neutral conditions, with object-based cues yielding a modest advantage. Neurally, both formats elicited robust modulations of induced alpha-band (8 to 14 Hz) activity distributed across visual, frontoparietal control, and memory-related networks. These effects followed a common temporal progression from early weak modulation to mid-latency reconfiguration and sustained late activity. However, object-based cueing produced more sustained and spatially widespread alpha suppression than feature-based cueing, indicating format-dependent differences in the dynamics of internal selection. Although neural effects closely tracked behavioral benefits at the condition level, no reliable brain–behavior correlations were observed across individuals. Together, these findings suggest that internal attention operates through a shared large-scale control architecture while flexibly adapting its temporal dynamics to representational format, with alpha-band activity playing a central role in the prioritization of WM content.

## Introduction

Imagine keeping a shopping list in mind—apples, milk, bread, and coffee. When you step into the fruit aisle, your mind immediately highlights “apples,” while the other items fade temporarily into the background. This everyday moment illustrates internal attention: the ability to selectively prioritize information held in working memory (WM). WM refers to the limited-capacity system that allows us to hold and manipulate information over short periods (Cowan 2001; Baddeley 2012). Understanding how internal attention interacts with WM is essential, as it governs which stored information is prioritized to guide behavior, supporting flexible adaptation to changing goals and environments (Van Ede and Nobre 2023).

Numerous studies have examined how external attention gates sensory signals and contributes to the formation of WM representations during encoding (Deubel and Schneidert 1996; Driver and Spence 1998; Busse et al. 2005; Wang et al. 2016, 2017; Schneegans and Bays 2017; London and Slagter 2020). More recently, increasing attention has been devoted to internal attention: how attention operates within WM to select, prioritize, and manipulate mental representations after initial encoding (Chun et al. 2011; Souza and Oberauer 2016; Rafi et al. 2023). This process is commonly studied using the retro-cue paradigm (Griffin and Nobre 2003; Landman et al. 2003), in which a cue presented after encoding indicates which information in memory is most likely to be tested. Such cues selectively enhance prioritized representations, producing the so-called retro-cue benefit.

Retro-cue studies have investigated different targets of internal selection (Lepsien and Nobre 2007; Kuo et al. 2009; Heuer and Schubö 2016; Poch et al. 2017; Heuer and Rolfs 2022), and how attention can reorder, filter, or inhibit encoded items to optimize performance (Bengson et al. 2020; Serin and Günseli 2022; Liu et al. 2025). Together, this research demonstrates that internal attention supports flexible, goal-directed behavior by dynamically prioritizing the most relevant information in WM (Atkinson et al. 2018; Van Ede et al. 2020; Nasrawi and van Ede 2022).

Neuroimaging research has associated internal attention with a general frontoparietal network involved in top-down control (Nee and Jonides 2009; Cisek and Kalaska 2010; Kia and Mesulam 2014; Lückmann et al. 2014; Chatham and Badre 2015), along with additional recruitment of cingulo-opercular regions implicated in sustained control and output gating (Spitzer and Blankenburg 2011; Wallis et al. 2015; Han et al. 2023). Temporally, internal selective attention was proposed to include 4 stages: orienting, selecting, prioritizing, and transforming (Myers et al. 2017; Van Ede and Nobre 2023).

However, this influential body of work has focused predominantly on object-based internal attention, where cues direct attention to a specific item stored in WM. While highly informative, such object-centered frameworks may not fully capture the broader landscape of internal attentional processes. For example, these studies frame internal selection within a spatial scaffold (Kuo et al. 2009; Groen et al. 2022), suggesting that space provides a fundamental structure/mediation for organizing working-memory representations (Qian et al. 2020; Yousif et al. 2021), but also see Sone et al. (2021). Yet, this spatial emphasis may partly reflect the structure of object-based representations, where spatial-item binding is integral (Treisman and Zhang 2006), rather than a universal property of internal selection.

This raises a central question: Is internal attention supported by a unified, domain-general mechanism, or a flexible set of operations tailored to the format of the selected information? To address this, we contrasted object-based cuing, which directs attention to a specific item, with feature-based cuing, which targets a specific feature across items (Yeh et al. 2007; Heuer and Schubö 2016; Adam et al. 2020). Feature-based internal attention operates by prioritizing a specific attribute such as color, orientation, or motion direction across all stored items, rather than selecting any single item as a whole. It thus acts at the level of representational dimensions, allowing attention to be less reliant on spatial information.

Both object- and feature-based cues enhance WM performance (feature based: Pilling and Barrett 2016; Ye et al. 2016, 2021; Kalogeropoulou et al. 2017; Niklaus et al. 2017; Park et al. 2017; Lee and Geng 2020), yet object-based cues often yield stronger behavioral benefits (Lin et al. 2021) and are associated with distinct patterns of alpha-band modulation (Hajonides et al. 2020). These divergences suggest that internal attention may not be a monolithic process but could instead engage format-specific neural operations.

However, whether internal attention implements different mechanisms across cue formats, or instead relies on a common underlying process, remains an open gap in the literature. To directly address this gap, we compared the neural dynamics of feature-based and object-based retro-cueing using magnetoencephalography (MEG), which offers high temporal resolution and reasonable spatial resolution, providing a powerful window into the timing and cortical distribution of internal selection processes. Notably, although several MEG studies have examined spatial (and thus object-based) retro-cues, typically focusing on alpha-band modulations (Poch et al. 2014, 2017; Wallis et al. 2015; Mok et al. 2016), no MEG work has investigated feature-based internal attention. By tracking the spatiotemporal patterns of cue-related alpha-band modulations of 2 kinds of retro-cues, we aimed to determine how internal attention flexibly operates across different cue formats.

## Materials and methods

### Participants

Forty-six adults (33 females; mean age 28, range 19 to 48) participated, all fluent in Finnish. Each visited the lab twice: the first visit involved 2 MEG tasks (feature and object cue tasks; order counterbalanced), and the second included unrelated cognitive tasks. The study was approved by the University of Jyväskylä ethics committee with diary number: 1376/13.00.04.00/2022, conducted in accordance with the Declaration of Helsinki. All participants provided written informed consent, and they received a 15-euro gift card or movie ticket.

Due to participant fatigue and technical problems, 2 feature cue datasets were unrecorded or lost, leaving 46 participants for object cue behavioral analyses and 44 for feature cue. One MEG dataset was excluded due to metal artifacts at the sensor level, and additional exclusions for missing empty-room recordings yielded 39 valid feature cue and 41 object cue source-level datasets. To ensure consistency across tasks, a common subset of 39 participants was used for source-level analyses.

Post hoc power analyses (G*Power 3.1) indicated that *N* = 39 provided 80% power (*α* = 0.05) to detect medium effects for paired t-tests (*dz* = 0.46) and repeated-measures analyses of variance (ANOVAs) (*f* ≈ 0.25, within-subject, *r* = 0.5), confirming adequate sensitivity to detect condition differences in cue-related neural dynamics.

### Experimental procedures

**Figure 1:**
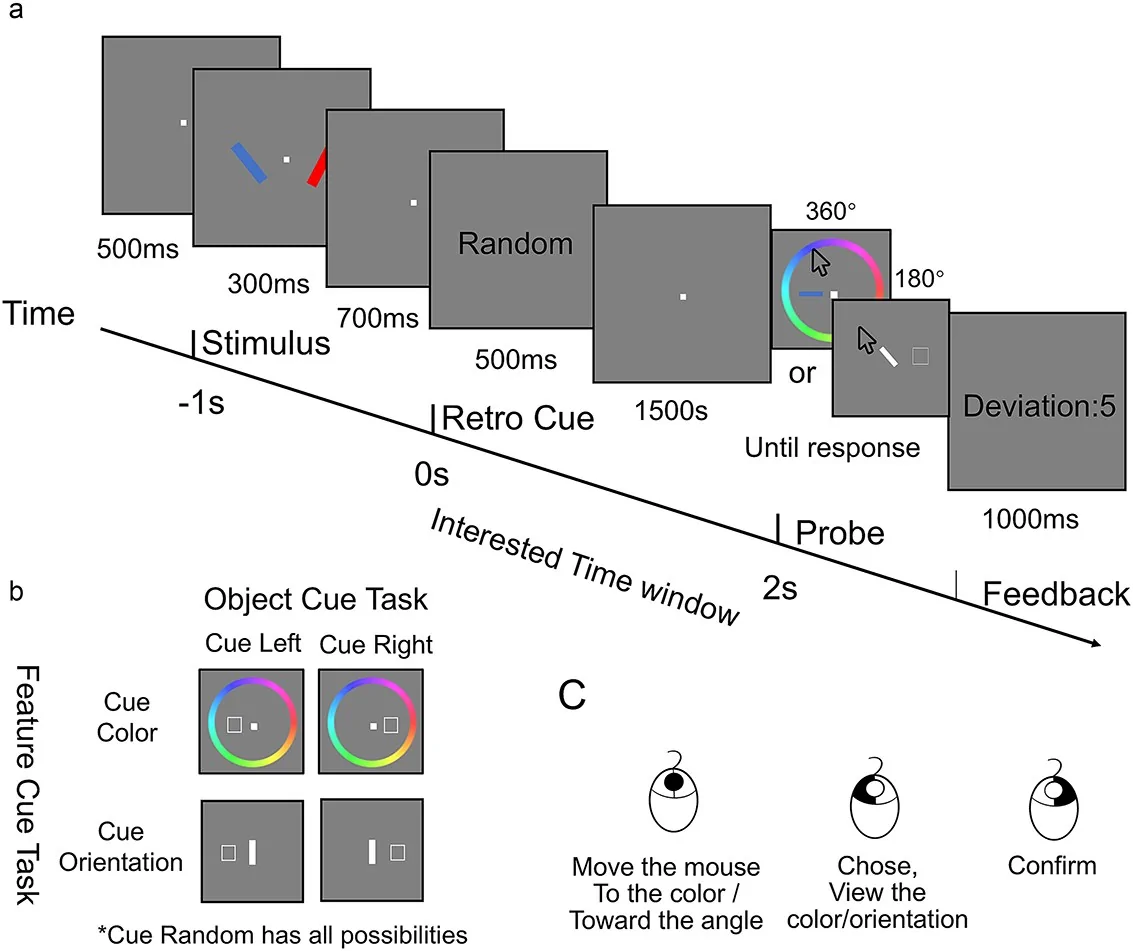
Experiment paradigm and probe information. The experiment included 2 tasks with a shared paradigm. a) The feature cue task and the object cue task. In the feature cue task, cues specified the feature to be recalled (color, orientation, or random), which indicated by the color ring/middle bar. In the object cue task, cues specified the object to be recalled (left, right, or random), which indicated by the left/right hollow square. The panel illustrates all possible probe types across conditions b) and the general sequence of operations c) during the probe period.

The experiment comprised 2 tasks: a feature cue task (with color, orientation, and neutral conditions) and an object cue task (with left, right, and neutral conditions), all conditions randomly intermixed within task, yielding 6 conditions that differed only in cue and probe type (Fig. 1). Each trial began with a 500 ms fixation, followed by a 300 ms memory array, 700 ms fixation, a 500 ms cue, and 1,500 ms fixation, after which the probe remained until a right-hand mouse response (left button to select, right to confirm) and 1,000 ms feedback. To ensure consistent processing, all cues were 100% valid, tasks were run in separate sessions with pseudo-randomized order, and participants practiced until reaching <10 deviation. Each task included 4 blocks of 60 trials (20 per condition), totaling 240 trials, with ~10 min per block, 15 min rest between tasks, and an overall MEG session of ~95 min.

### Stimuli

Both MEG tasks used identical visual stimuli but differed in the word cues directing attention (Fig. 1). Each trial comprised 4 phases: stimulus, retro-cue, probe, and feedback, following Ye et al. (2016) with Finnish word cues. During the stimulus phase, 2 bars (1 per side of a central fixation) displayed color (from a 360° space) and orientation (from 180° angles). In the retro-cue phase, the feature task used the word “Suunta” (orientation), “Väri” (color), or “Random” (neutral) as a cue; the object task used the word “Vasen” (left), “Oikea” (right), or “Random” (neutral) as a cue. During the probe, a square indicated the probed object, and participants reported via mouse—using a color wheel for color recall or a rotatable bar for orientation recall. Feedback displayed “erotus” (deviation) and a number, with lower values indicating higher accuracy (0 = perfect, 180/90 = worst).

### Recordings

MEG data were recorded using a 306-channel Elekta Neuromag system (MEGIN Oy, Helsinki, Finland) inside a magnetically shielded room (Vacuum Schmelze GmbH, Hanau, Germany) at the Center for Interdisciplinary Brain Research, University of Jyväskylä. Each task comprised 4 MEG recording blocks, yielding a total of 8 recordings per participant, along with additional empty-room recordings for noise estimation. The MEG signals were band-pass filtered at 0.1 to 330 Hz and sampled at 1,000 Hz. Head position was continuously tracked using 5 head position indicator coils. Two pairs of electrodes were used to record electrooculography: 1 pair next to right and left eyes and the other pair below and above the right eye. A pair of electrodes placed approximately 5 cm below the collarbone served to monitor the electrocardiograph for heartbeat detection. The ground electrode was placed on the right collarbone. Prior the MEG data recording, 3 anatomical landmarks, including the nasion, as well as left and right preauricular points, were recorded together with over 200 scalp points to establish the head coordinate frame.

### Behavioral data analysis

To account for differences in physical scales between color and orientation probes, behavioral performance was quantified as the mean absolute angular deviation between reported and target orientations. This error was scaled relative to the maximum possible angular deviation for each feature type (color: 180°, orientation: 90°), yielding a comparable bounded measure ranging from 0 to 1.


$$\mathrm{Normalized}\ \mathrm{Error}=\frac{\left|{\theta}_{\mathrm{response}}-{\theta}_{\mathrm{target}}\right|}{\theta_{\mathrm{max}}}$$

Retro-cue benefits were first confirmed using paired t-tests of normalized error value comparing informative cue trials with probe-matched neutral (random) trials within each task. We then quantified retro-cue benefits as the normalized error difference between informative-cue and probe-matched neutral trials for each task. These cueing benefits were compared across cue types (color vs. orientation; left vs. right), and subsequently averaged to contrast overall feature- versus object-based attention effects. Baseline performance in the random conditions of the 2 tasks was also directly compared to ensure comparable task difficulty. This analysis provides a simple and interpretable summary of behavioral performance without relying on multiparameter mixture-model fits (eg Zhang and Luck 2008), which are informative but not essential for the present MEG-focused investigation. To further validate the normalization assumption, raw error distributions across conditions are provided in Fig. S1.

### MEG data analysis

#### Preprocessing

MEG data were preprocessed using a standardized pipeline. All analyses were performed using MNE (Magnetoencephalography- and Electroencephalography-Python; MNE-Python) (Gramfort et al. 2013) and general-purpose Python tools. First, the Oversampled Temporal Projection was applied to suppress sensor noise and enhance signal quality for all 8 blocks of recordings. The Maxfilter algorithm (Elekta Oy) was used to apply temporal Signal Space Separation with the head-movement compensation option to correct for head movements, remove external magnetic interference, and interpolate noisy channels. To ensure consistent head alignment across blocks, the mean head position across all 8 recordings was computed for each participant, and individual blocks were transferred to this mean position. Afterward, recordings within each task were concatenated, resampled to 250 Hz, and band-pass filtered between 1 and 100 Hz to further reduce data size and focus on relevant frequency components. Finally, independent component analysis was used to identify and automatically remove artifacts caused by eye blinks and cardiac signals.

#### Source analysis

For each condition, epochs were extracted from −2 to 2.5 s relative to cue onset. Epochs with higher amplitude than 40,000 fT (magnetometer) or 3,000 fT/cm (gradiometer), or lower than flat criteria (1 fT in magnetometers or 1 fT/cm for gradiometers) were excluded. The evoked response was removed prior to time–frequency decomposition to minimize phase-locked cue-evoked activity that could contaminate oscillatory estimates near cue onset. Time–frequency representations (TFRs) were then computed using Morlet wavelets from 3 to 45 Hz, with a fixed number of 5 cycles per frequency (balancing temporal and spectral resolution). The resulting TFRs were cropped to a −0.5 to 2 s time window. For visualization, TFRs were first averaged across participants and baseline-corrected using the −0.5 to 0 ms interval. MEG channels were then grouped into 8 areas—frontal, occipital, parietal, and temporal in each hemisphere—based on the spatial distribution of sensor coverage. Although no statistical analyses were performed at the sensor level, Also, contrast TFRs were obtained by subtracting each cued condition from its corresponding random condition, yielding 4 contrasts: color-random and orientation-random (feature cue task), and left-random and right-random (object cue task).

Visualization of time–frequency heatmaps showed a consistent oscillatory pattern across all 6 conditions, mainly with increased alpha power over occipital and parietal sensors (Fig. S2). Then, the visualization of time–frequency contrasts concerning 4 cue-versus-random pairs showed that object cue conditions exhibited widespread alpha suppression throughout the trial compared to random conditions, whereas feature cue conditions displayed more divergent dynamics (Fig. S3).

Based on the TFR results and prior literature (Poch et al. 2014, 2017; Wallis et al. 2015; Mok et al. 2016; Hajonides et al. 2020), we focused on induced alpha power (8 to 14 Hz) within the time window of −0.5 to 2 s to examine widespread oscillatory modulations in the brain. As individual MRI scans were not available, we used the MNE standard average head model (fsaverage), which was co-registered to each participant’s digitized head shape. A boundary element model was constructed with a single-layer surface (conductivity = 0.3, ico = 4, mindist = 5 mm) to compute the forward solution. The noise covariance matrix was estimated using empty-room recordings obtained for each participant.

An inverse operator was then created using the following parameters: loose = “auto,” depth = 0.8, and use_cps = True, enabling anatomically constrained source estimation. Source estimates were computed using the standardized low-resolution brain electromagnetic tomography method with lambda2 = 1/9, separately for each TFR epoch. Baseline normalization was applied by computing source-level power within the pre-cue interval (−0.5 to 0 s), which was subtracted (in dB) from post-cue activity to highlight internal attention-induced alpha modulation. Although low-frequency time–frequency estimates may exhibit limited temporal smearing around cue onset, the use of evoked-response subtraction and identical preprocessing across conditions reduced the likelihood that this systematically biased the between-condition comparisons.

Source estimates were collapsed across the 8 to 14 Hz range and parcellated into 448 cortical regions using the fine-grained atlas by Khan et al. (2018) implemented in MNE-Python. This reduced the high-dimensional source space (8,196 vertices) to anatomically and functionally meaningful regions, improving signal-to-noise ratio and facilitating statistical analysis. Six source data matrices (1 per condition) were then constructed, each comprising 626 time points (−0.5 to 2 s), 448 regions, and data from 39 participants. Significant activation patterns relative to baseline for all 6 conditions are shown in Fig. S4.

#### Regions of interest definition

To characterize the spatiotemporal dynamics of internal attention, 5 bilateral large-scale functional networks were predefined based on previous literature implicating these systems in visual WM and attentional control (Nee and Jonides 2009; Wallis et al. 2015; Myers et al. 2017). All regions of interest (ROIs) were constructed as spatial masks derived from a 448-parcel cortical atlas used for source reconstruction (Khan et al. 2018), and were grouped based on spatial proximity and presumed functional coherence, ensuring anatomical interpretability and reproducibility (Fig. S5).

**Figure 2:**
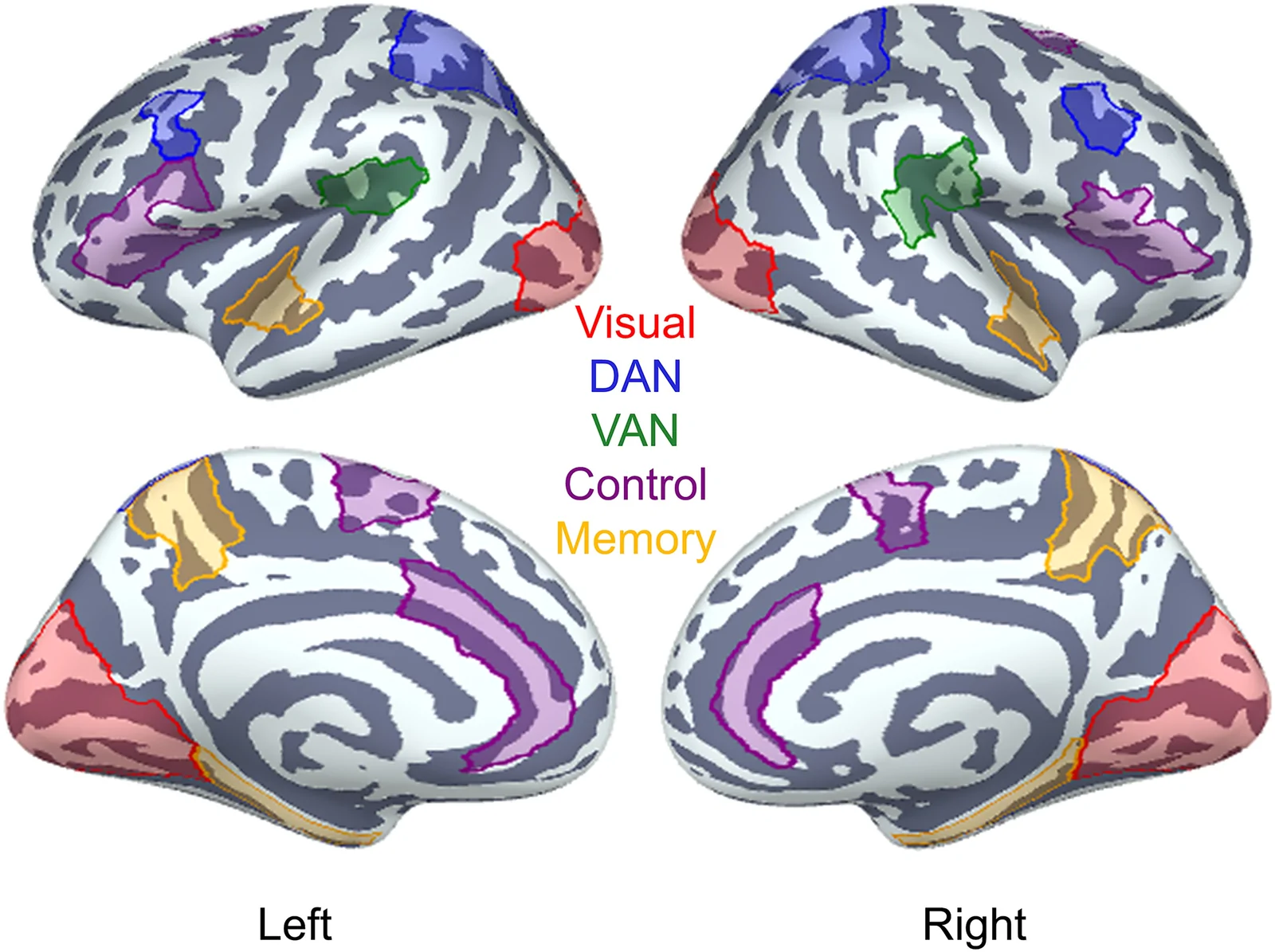
Regions of interest (ROIs) definition. ROIs were combined into 5 functional networks: visual, DAN, VAN, control, and memory-related regions. Each network is color-coded and rendered on the cortical surface (lateral and medial views, both hemispheres).

As shown in Fig. 2, these networks included: (i) visual regions encompassing the occipital cortex, (ii) the dorsal attention network (DAN; including the superior parietal cortex and frontal eye fields), (iii) the ventral attention network (VAN; including the temporoparietal junction), (iv) the fronto-cingulate control network comprising the anterior cingulate cortex, supplementary motor area, and inferior frontal gyrus, and (v) a memory-related/internal representation network including the precuneus, medial temporal lobe structures, and anterior temporal cortex.

ROIs were defined bilaterally and aggregated at the network level to capture large-scale functional dynamics. This approach allowed for a structured examination of interactions among posterior sensory, attentional control, and internal representation systems during internal attention.

### Statistical analysis

For each participant and condition, time courses were computed over the interval from −0.5 to 2 s relative to cue onset. Statistical analyses focused on post-cue activity (0 to 2 s). To characterize condition-specific activation patterns, each cued condition was compared against its corresponding random baseline (eg color vs. random, orientation vs. random, left vs. random, and right vs. random) using nonparametric cluster-based permutation tests.

To quantify cueing benefits, difference waves were computed by subtracting the random condition from each cued condition. For the feature-based task, cueing benefit was defined as the average of color—random and orientation—random, whereas for the object-based task, it was defined as the average of left—random and right—random.

To assess differences between cue types within each task, paired comparisons were performed between individual cueing benefits (eg color—random vs. orientation—random; left—random vs. right—random).

Finally, to directly compare attentional formats, cueing benefits were contrasted between tasks (feature vs. object).

All statistical comparisons were performed using nonparametric cluster-based permutation tests (2-tailed), as implemented in MNE-Python, controlling for multiple comparisons across time points. Cluster significance was determined based on a permutation distribution (2000 permutations), with clusters considered significant at *P* < 0.05.

### Frequency specificity and brain–behavior correlation analysis

Motivated by time–frequency visualizations, identical analyses were additionally performed in the theta (4 to 7 Hz) and beta (15 to 25 Hz) bands, using the same preprocessing, source reconstruction, and statistical procedures as for the alpha-band analysis.

To examine the relationship between neural activity and behavior, brain–behavior associations were assessed in the alpha band (8 to 14 Hz). A time-resolved general linear model (GLM) was applied at each time point, relating individual differences in neural cueing effects to behavioral cueing benefits for each ROI. Statistical significance was evaluated using nonparametric cluster-based permutation testing (2,000 permutations, 2-tailed), controlling for multiple comparisons across time. Clusters were considered significant at a cluster-level threshold of *P* < 0.05.

## Results

### Behavioral results

**Figure 3:**
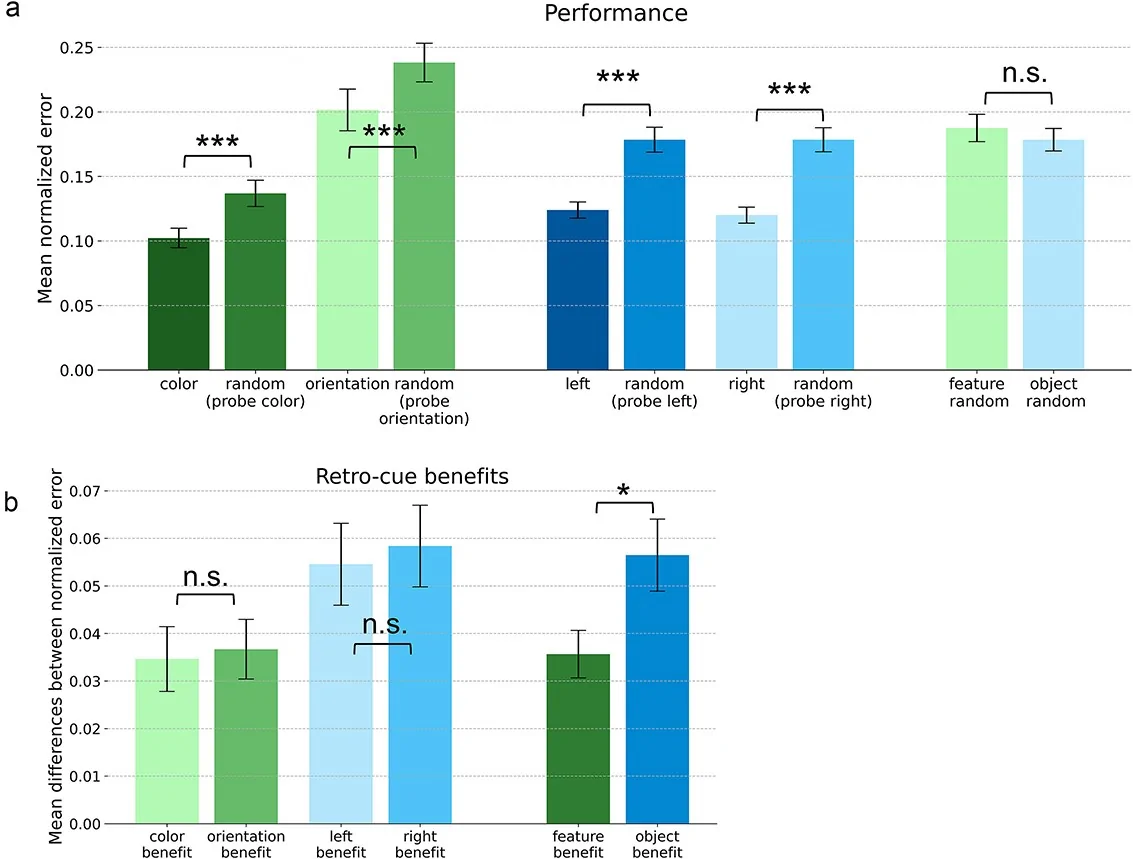
Behavioral results. Error bars represent the SEM. a) Performance across all cue conditions and the corresponding test trials from the random condition. b) Retro-cue benefits within the feature cue task and the object cue task, as well as the overall retro-cue benefit for 2 tasks.

Indicated by normalized error, task performance (Fig. 3a) in cue conditions was better than in matched random probe trials for both the feature cue task (color vs. random probe color: *t*(43) = −5.077, *P* < 0.001, Cohen’s *d* = 0.77, 95% CI [−1.10, −0.43]; orientation vs. random probe orientation: *t*(43) = −5.855, *P* < 0.001, Cohen’s *d* = 0.88, 95% CI [−1.23, −0.53]) and the object cue task (left vs. random probe left: *t*(45) = −6.331, *P* < 0.001, Cohen’s *d* = 0.93, 95% CI [−1.28, −0.58]; right vs. random probe right: *t*(45) = −6.805, *P* < 0.001, Cohen’s *d* = 1.00, 95% CI [−1.36, −0.64]). No significant difference was found between the random conditions of the 2 tasks (*t*(43) = 1.268, *P* = 0.212, Cohen’s *d* = −0.19, 95% CI [−0.11, 0.49]), indicating comparable baseline performance.

Then for the differential benefit of cue types (Fig. 3b), no significant difference was found between the color and orientation cue benefit in the feature cue task (*t*(43) = −0.244, *P* = 0.808, Cohen’s *d* = 0.04, 95% CI [−0.33, 0.26]), nor between left and right cues in the object cue task (*t*(45) = −0.475, *P* = 0.637, Cohen’s *d* = −0.07, 95% CI [−0.36, 0.22]). However, overall retro-cue benefits were significantly greater in the object cue task compared to the feature cue task (*t*(43) = −2.051, *P* = 0.046, Cohen’s *d* = −0.31, 95% CI [−0.61, −0.01]), suggesting that object-based cues were more effective in enhancing behavioral performance than feature-based cues.

### Alpha-band dynamics

Across all conditions, retro-cues induced robust modulations of induced alpha-band power (8 to 14 Hz) relative to pre-cue baseline. These modulations were distributed across large-scale cortical networks, including visual, DAN, VAN, fronto-cingulate control, and memory-related networks.

A consistent temporal structure emerged across conditions, characterized by minimal early effects (0 to 0.4 s), pronounced modulation during a mid-interval (0.4 to 0.9 s), and sustained activity during a late interval (0.9 to 2 s). This temporal progression formed the basis for subsequent condition comparisons.

### Cueing benefits relative to random baseline

**Figure 4:**
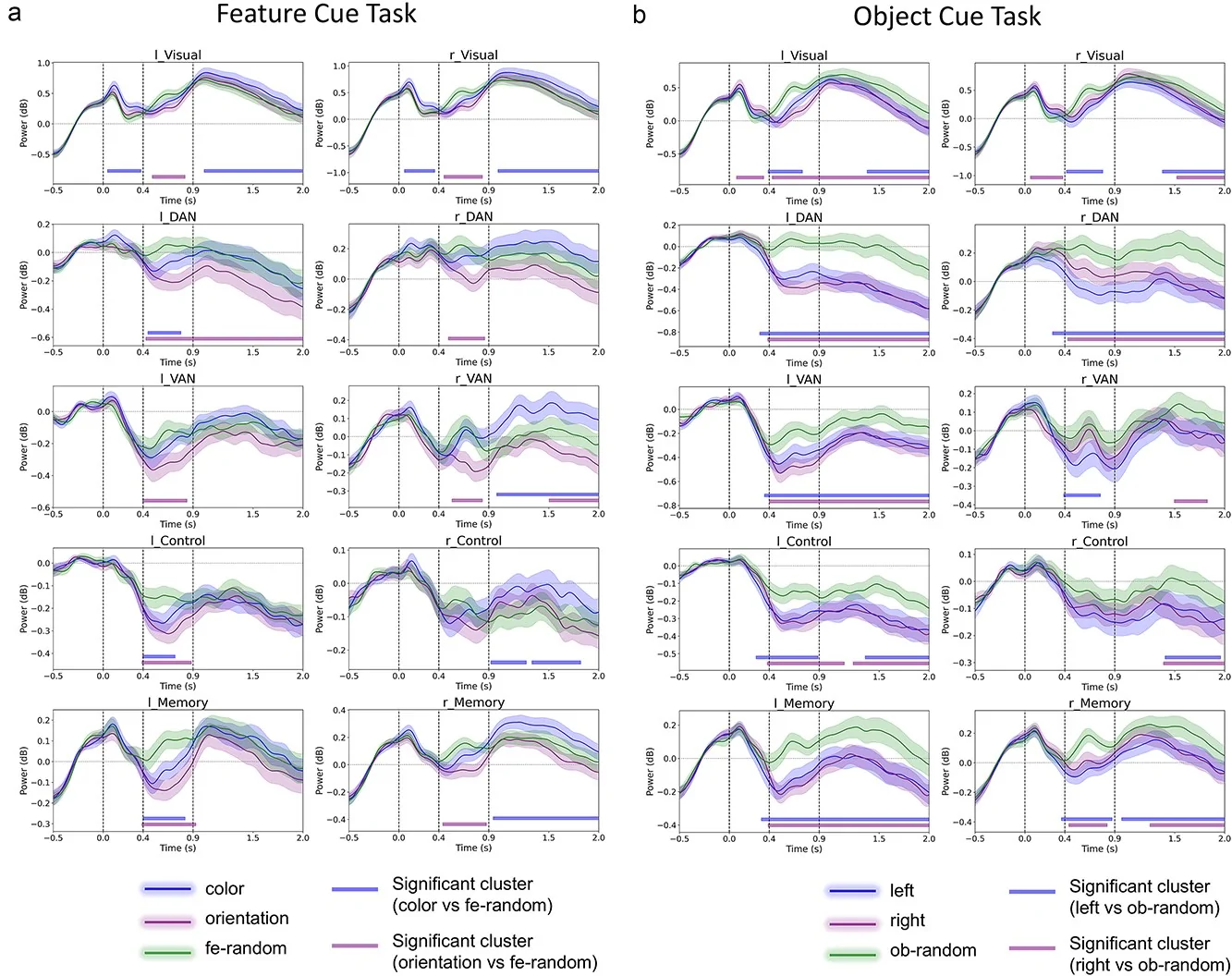
Cueing benefits relative to the random baseline in the alpha band (8 to 14 Hz). a) Time courses of alpha-band cueing effects in the feature cue task, shown as the difference between informative cue conditions and the random condition (color—random; orientation—random) across predefined cortical networks. b) Time courses of alpha-band cueing effects in the object cue task, shown as the difference between informative cue conditions and the random condition (left—random; right—random). Shaded regions represent ± SEM across participants. Horizontal bars indicate significant time clusters identified using nonparametric cluster-based permutation tests (2-tailed, cluster-level *P* < 0.05).

Relative to the random condition, color cues (Fig. 4a) elicited early (0 to 0.4 s) alpha increases in bilateral visual cortices, followed by transient suppression (0.4 to 0.9 s) in left DAN, VAN, control, and memory-related networks. During the late interval (0.9 to 2 s), alpha rebounded in bilateral visual cortices and in right VAN, control, and memory-related networks.

In contrast, orientation cues (Fig. 4a) showed no early modulation. Transient suppression (0.4 to 0.9 s) was observed in bilateral visual cortices, DAN, VAN, and memory-related networks, as well as in the left control network. This suppression persisted into the late interval in left DAN and right VAN.

In the object cue task, left and right cues elicited highly similar alpha dynamics (Fig. 4b), characterized by sustained suppression from approximately 0.4 to 2 s across all networks.

### Within-format comparisons

**Figure 5:**
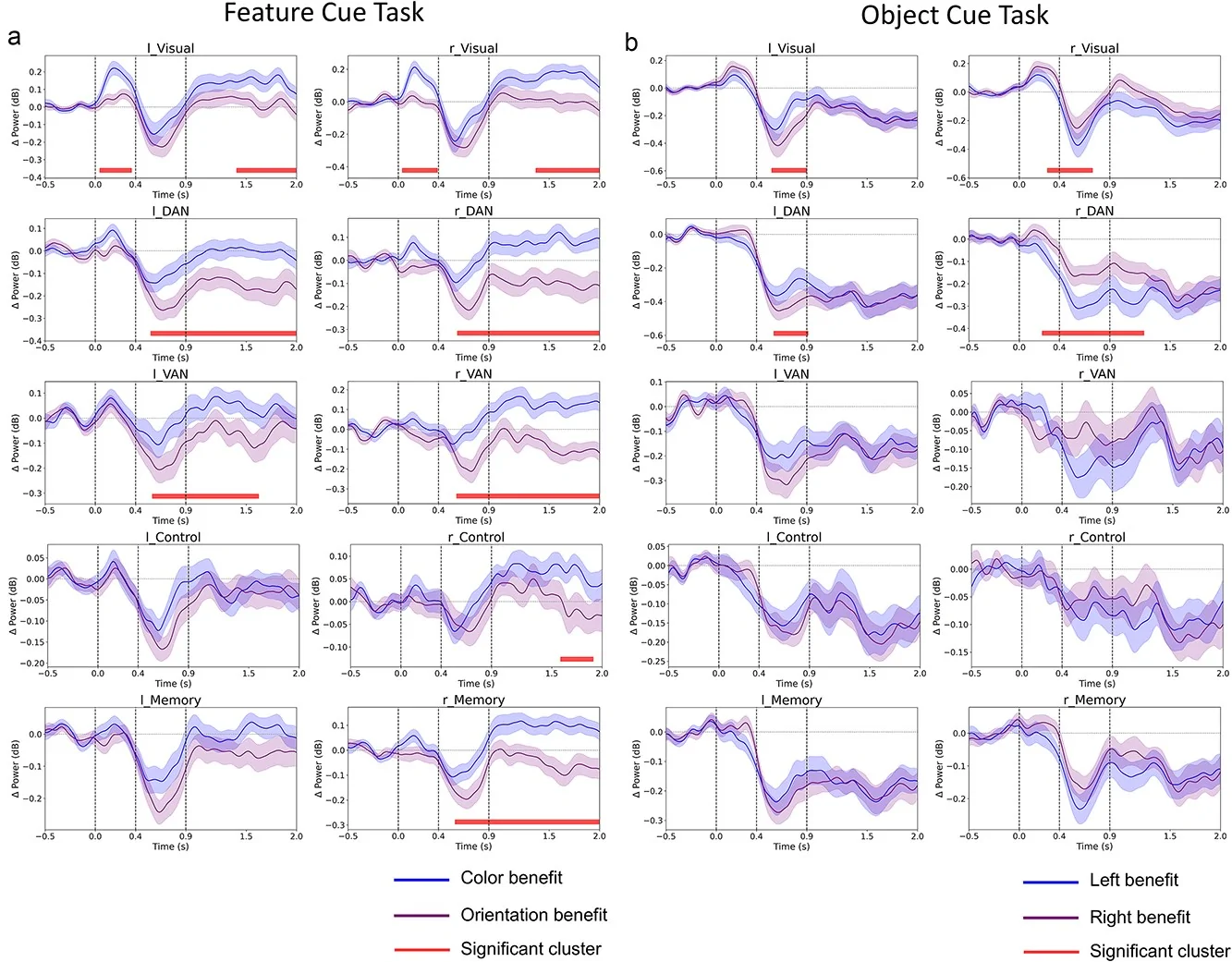
Within-format comparisons of alpha-band cueing dynamics across cortical networks. a) Time courses comparing feature-based cueing effects between color and orientation cues, expressed as alpha-band (8 to 14 Hz) power modulation relative to the corresponding random condition. b) Time courses comparing object-based cueing effects between left and right cues. Shaded regions represent ± SEM across participants. Horizontal bars indicate significant time clusters identified using nonparametric cluster-based permutation tests (2-tailed, cluster-level *P* < 0.05).

Within the feature cue task, color and orientation cues elicited partially overlapping but distinct alpha dynamics (Fig. 5a). Overall, orientation cues showed stronger suppression than color cues across the time course. Differences were observed during early and late intervals in visual cortices, during mid-to-late intervals in bilateral DAN, VAN, and right memory-related networks, and during the late interval in the right control network.

Within the object cue task, left and right cueing benefits were largely similar (Fig. 5b). A lateralized pattern was observed in VAN and DAN during the mid-time interval, with stronger contralateral suppression relative to the cued side.

### Cross-format comparison (feature vs. object)

**Figure 6:**
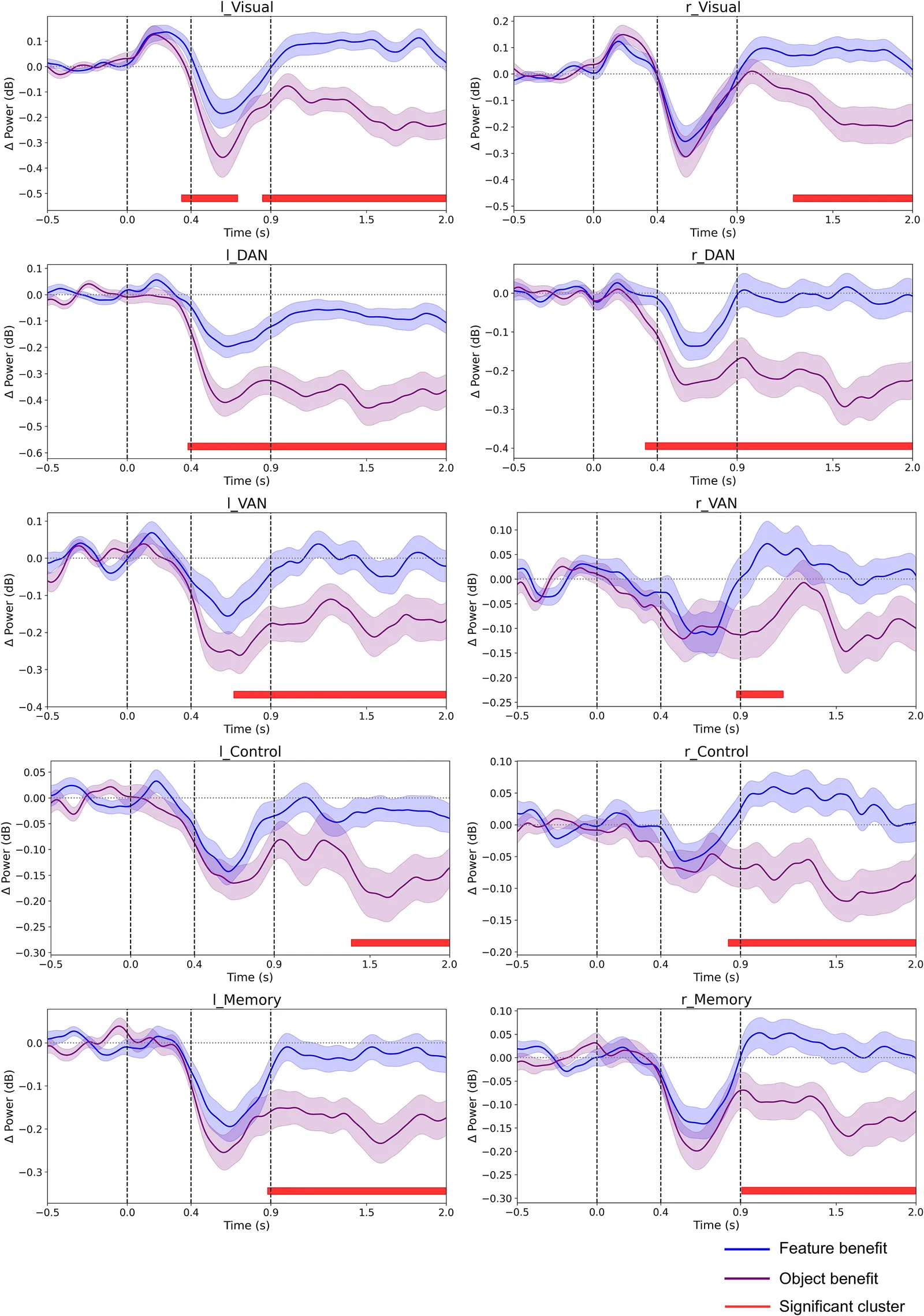
Cross-format comparison of alpha-band cueing effects between feature-based and object-based attention. Time courses show differences in alpha-band (8 to 14 Hz) cueing benefits between object-based and feature-based conditions, computed as the contrast between the 2 tasks across predefined cortical networks. Significant time clusters were identified using nonparametric cluster-based permutation testing (2-tailed, cluster-level *P* < 0.05). Shaded regions represent ± SEM across participants.

When comparing cue-related benefits across 2 tasks, we found that object-based cueing elicited a more sustained pattern of alpha suppression than feature-based cueing (Fig. 6). Specifically, during the mid-time window, significant differences were observed in the left visual cortex, the left VAN, and bilateral DAN. In the late time window, the benefit associated with object cues was reflected in deeper alpha suppression across all networks.

### Frequency specificity and brain–behavior relationships

To assess whether format-dependent effects generalized beyond the alpha band, identical analyses were conducted in the theta (4 to 7 Hz) and beta (15 to 25 Hz) bands.

No significant differences between feature- and object-based cueing were observed in the theta band (see Fig. S6). In contrast, beta-band activity showed partial format-dependent modulation. These effects emerged in limited time windows and regions but were weaker and less temporally sustained compared to those observed in the alpha band (see Fig. S7).

To further examine the functional relevance of alpha-band dynamics, brain–behavior relationships were assessed using a time-resolved GLM combined with cluster-based permutation testing. Although descriptive fluctuations in correlation coefficients were observed over time (see Fig. S8), no significant clusters were identified after correction for multiple comparisons (all cluster-level *P* > 0.05).

## Discussion

The present study provided converging evidence that internal attention flexibly prioritizes WM representations depending on cue format. Behaviorally, retro-cues improved performance across both feature-based and object-based tasks, replicating the well-established retro-cue benefit. At the neural level, these effects were primarily expressed in alpha-band (8 to 14 Hz) dynamics, with object-based cueing producing stronger and more sustained benefits than feature-based cueing. Together, these findings suggest that while internal attention operates as a general mechanism for prioritizing memory representations, its efficiency and neural implementation depend on the format of the selected information.

### Shared and distinct neural dynamics of internal attention

Across all conditions, internal attention was associated with a consistent temporal progression of alpha-band modulation, beginning with modest and relatively focal early changes, followed by pronounced mid-latency modulation and sustained late activity. These dynamics were distributed across large-scale cortical systems, including visual, dorsal, and ventral attention, fronto-cingulate control, and memory-related networks. This pattern aligns with previous proposals that internal attention unfolds as a multistage process involving orienting, selection, (re)prioritizing, and transforming (Myers et al. 2017; van Ede, F., and Nobre, A. C., 2024 ).

Some limited lateralized effects were observed in specific time windows; however, they were not consistent across conditions and are unlikely to reflect robust hemispheric specialization. Instead, these effects may be driven by task-related factors, including response mapping, motor preparation, or decision-related processes. In particular, the use of a fixed right-hand response across all right-handed participants may have introduced lateralized motor activity.

Despite these shared features, clear differences emerged between cue formats. Object-based cueing elicited robust and sustained alpha suppression across networks, which may reflect engagement of attentional control-related processes and sustained prioritization of selected representations (Jensen and Mazaheri 2010; Poch et al. 2014, 2017). In contrast, feature-based cueing showed more heterogeneous dynamics, including transient suppression and rebound increases in alpha power. These differences indicate that internal attention does not operate as a uniform process but instead adapts its neural dynamics depending on the structure of the selected information.

Importantly, these format-dependent effects were largely specific to the alpha band. No reliable differences were observed in theta activity, and only limited and transient effects emerged in the beta band. This pattern is consistent with the view that alpha oscillations provide a reliable index of internal attentional modulation, supporting the prioritization and functional regulation of WM representations (Myers et al. 2017; Van Ede and Nobre 2023).

While alpha-band modulations provide a reliable marker of condition-dependent neural dynamics, the present data do not allow a definitive mapping between specific temporal components and distinct underlying cognitive operations. In particular, early post-cue effects likely reflect a combination of stimulus-evoked responses and early task-related processing dynamics, rather than a process-specific signature of attentional selection.

### Format-dependent efficiency of internal attention

A key finding of the present study is that object-based cueing yielded stronger behavioral benefits and more sustained neural modulation than feature-based cueing. One interpretation is that object-based cues provide a more efficient format for internal selection. Because object representations integrate multiple features into a unified entity, prioritizing an object may facilitate more stable prioritization of task-relevant information (Treisman and Zhang 2006; Zhang and Luck 2008; Woodman et al. 2022).

In contrast, feature-based cueing requires selecting a specific dimension across multiple items, which may involve more distributed and potentially competing representations (Heuer and Schubö 2016; Adam et al. 2020). This could lead to less stable prioritization, reflected in the more variable alpha dynamics observed here. From this perspective, differences between cue formats may reflect variations in the efficiency with which internal attention can isolate and maintain relevant information, rather than entirely distinct underlying mechanisms.

However, these interpretations should be treated with caution. Notably, we did not observe significant brain–behavior correlations linking alpha modulation to individual differences in behavioral benefits. This suggests that while alpha dynamics reliably track condition-level effects, their direct functional contribution to performance remains unresolved.

### Format-specific operations within feature-based attention

Within the feature-based task, color and orientation cues exhibited partially overlapping but distinct neural dynamics. Color cues were associated with an early increase in alpha power followed by suppression and late rebound, whereas orientation cues elicited more sustained suppression. These differences suggest that feature-based attention is not a unitary process but depends on the specific representational format of the selected feature (Hajonides et al. 2020; Slana Ozimič et al. 2023).

One possibility is that different features place distinct representational demands on internal attention. For example, color representations may rely more strongly on sensory-perceptual coding, whereas orientation representations may engage stronger spatial or relational constraints (Qian et al. 2020; Yousif et al. 2021). Such differences could shape how internal attention operates on these representations, leading to distinct temporal profiles of alpha modulation.

In contrast, object-based cueing produced largely homogeneous dynamics across left and right cues, with only limited lateralized effects. This further supports the idea that object-based attention operates on integrated representations (Treisman and Zhang 2006; Zhang and Luck 2008), resulting in more consistent neural patterns across conditions.

### Limitations and future directions

Several limitations should be considered. First, source reconstruction relied on a standard template (fsaverage) rather than individual anatomical MRI scans, which may reduce spatial precision. Second, the absence of significant brain–behavior correlations limits our ability to directly link neural dynamics to behavioral performance. Third, the present study focused primarily on oscillatory power, leaving open questions regarding how prioritized memory representations are coordinated across cortical networks and represented over time. In addition, because low-frequency time–frequency estimates can exhibit temporal smearing around cue onset, subtle theta-band effects may have been underestimated despite the use of evoked-response subtraction and conservative preprocessing procedures.

Future work could address these limitations by combining MEG with multivariate decoding and connectivity-based approaches to better characterize the dynamic organization of internal attention (Stokes 2015; Grootswagers et al. 2017; de Vries et al. 2019; Van Ede and Nobre 2023). In addition, examining a broader range of feature dimensions and incorporating causal manipulations, such as transcranial magnetic stimulation, may help clarify how representational format shapes attentional prioritization in WM.

## Conclusion

In summary, the present study demonstrates that internal attention operates as a flexible mechanism for prioritizing WM representations, but its efficiency and neural implementation depend on the format of the selected information. Using MEG, we showed that both feature-based and object-based retro-cues reliably enhanced behavioral performance and modulated large-scale alpha-band dynamics. However, object-based cueing produced stronger behavioral benefits and more sustained alpha suppression across cortical networks, whereas feature-based cueing exhibited more heterogeneous and temporally variable patterns.

These findings suggest that internal attention is not a unitary process, but is shaped by the representational structure of WM content. Rather than relying on entirely distinct mechanisms, different cue formats may engage a common attentional system with varying efficiency depending on how information is organized and accessed.

More broadly, our results highlight the importance of considering representational format when studying internal attention, and provide new evidence that alpha-band dynamics appear to play a key role in coordinating the prioritization of information in WM.

## Supplementary Material

supplementary-final_bhag110

## Data Availability

The datasets generated and/or analyzed during the current study are not publicly available due to ethical restrictions and lack of participant consent, but the analysis code is available at https://osf.io/vxpsb/files/osfstorage.
