# Supplementary material for: Flexibility of internal attention: divergent spatiotemporal profiles for feature and object cueing
Source: Cereb Cortex. 2026 Sep 17;36(9):bhag110. doi: 10.1093/cercor/bhag110 (PMC13584068; doi:10.1093/cercor/bhag110)
Supplement: Supplementary file 1 [file supplementary-final_bhag110.docx]

**Supplementary Materials**

**Flexibility of Internal Attention: Divergent Spatiotemporal Profiles for Feature and Object Cueing**

Xiaoshu Lin^1 2^; Kristina Pultsina^1 2^; Simo Monto^1 2^; Chaoxiong Ye^13*^; Tiina Parviainen^1 2*^

1 Department of Psychology, University of Jyväskylä, PO Box 35, Jyväskylä FI-40014, Finland

2 Centre for Interdisciplinary Brain Research, University of Jyväskylä, PO Box 35, Jyväskylä FI-40014, Finland

3 School of Education, Anyang Normal University, Anyang, 455000, China

*Corresponding should be addressed to Tiina Parviainen, Centre for Interdisciplinary Brain Research, University of Jyväskylä, PO Box 35, Jyväskylä FI-40014, Finland, email: [tiina.m.parviainen@jyu.fi](mailto:tiina.m.parviainen@jyu.fi), and Chaoxiong Ye, Department of Psychology, University of Jyväskylä, PO Box 35, Jyväskylä FI-40014, Finland, email: [cxye1988@163.com](mailto:chaoxiong.c.ye@jyu.fi)

Figure S1. Distribution of raw behavioral errors across experimental conditions.

Violin plots show the distribution of trial-level raw absolute recall errors (in degrees) across all participants for the feature cue task (left) and object cue task (right). Black dots represent individual trials. Error values are shown prior to normalization and correspond to the absolute angular deviation between the reported and target feature values.

Figure S2. Time-Frequency Representation (TFR) across six conditions. Each condition includes eight TFRs derived from the left and right frontal, occipital, parietal, and temporal channel groups. Induced responses are displayed on a uniform scale ranging from –3×10⁻²³ to 3×10⁻²³.

Figure S3. Contrast Time-Frequency Representation (TFR) across four pairs.

Each pair includes eight TFRs derived from the left and right frontal, occipital, parietal, and temporal channel groups. Induced responses are displayed on a uniform scale ranging from –8 × 10⁻²⁴ to 8 × 10⁻²⁴.

Figure S4. Source activations across six conditions.
Source-level data for each condition were extracted in eight consecutive 250 ms segments. Significant regions are shown on brain maps, where blue indicates decreases and red indicates increases in alpha power, using a uniform scale from –1 to 1.

Figure S5. Definition of functional regions of interest (ROIs).

Regions of interest were defined based on a 448-parcel cortical atlas and grouped into five bilateral functional networks (visual, dorsal attention, ventral attention, control, and memory-related systems). Parcels were aggregated based on anatomical proximity and functional coherence to enable large-scale network-level analyses of internal attention.

Figure S6. Theta-band (4–7 Hz) cueing effects in feature- and object-based attention.

Time courses show theta-band (4–7 Hz) cueing-related effects for feature-based and object-based conditions across predefined cortical networks. Effects were computed as the difference between each cued condition and the corresponding random baseline. No significant clusters were identified using nonparametric cluster-based permutation testing (two-tailed, cluster-level p < 0.05). Shaded regions represent ± SEM across participants

Figure S7. Beta-band (15–25 Hz) cueing effects in feature- and object-based attention.

Time courses show beta-band (15–25 Hz) cueing-related effects for feature-based and object-based conditions across predefined cortical networks. Effects were computed as the difference between each cued condition and the corresponding random baseline. Significant clusters were identified using nonparametric cluster-based permutation testing (two-tailed, cluster-level p < 0.05), with effects showing limited and transient spatiotemporal modulation relative to alpha-band dynamics. Shaded regions represent ± SEM across participants.

Figure S8. Time-resolved brain–behavior relationships in alpha-band activity across ROIs.

Time-resolved relationships between alpha-band (8–14 Hz) neural activity and behavioral cueing benefits were assessed across predefined cortical regions of interest (ROIs). The top panel shows condition-level differences between object-based and feature-based cueing (Δ power), with significant clusters identified using nonparametric cluster-based permutation testing (red bars, cluster-level p < 0.05). The middle panel shows the time course of the neural contrast (object − feature). The bottom panel shows time-resolved brain–behavior correlations (Pearson’s r) between neural cueing effects and behavioral benefits, with significance assessed using a time-resolved general linear model and cluster-based permutation testing. No significant clusters were observed after correction for multiple comparisons (all cluster-level p > 0.05). Shaded regions represent ± SEM across participants.
